# Does Sport Participation Protect Adolescents from Alcohol Consumption? A Scoping Review

**DOI:** 10.3390/ijerph20075417

**Published:** 2023-04-06

**Authors:** Bartłomiej Walczak, Anna Walczak, Sandra Tricas-Sauras, Jakub Kołodziejczyk

**Affiliations:** 1 Faculty of Applied Social Sciences and Resocialization, University of Warsaw, Nowy Świat 69, 00-297 Warsaw, Poland; 2 Faculty of Social Sciences, Christian Theological Academy in Warsaw, Broniewskiego 48, 01-771 Warsaw, Poland; 3 École de Santé Publique (CR5-CRISS) Social Approaches of Health, Campus Erasme, Université Libre de Bruxelles, CP 596, Route de Lennik 808, 1070 Brussels, Belgium; 4 Erasmus Hogeschool Brussel, BRUCHI Kennis Centrum, Laarbeeklaan, 121, Jette, 1090 Brussels, Belgium; 5 European Alcohol Policy Alliance, Eurocare, Rue Archimède, 17, 1000 Brussels, Belgium; 6 Faculty of Management and Social Communication, Jagiellonian University in Cracov, Łojasiewicza 4, 30-348 Cracov, Poland

**Keywords:** alcohol, adolescents, sport participation, team sports, gender

## Abstract

(1) Background: Participation in youth sports is believed to protect against alcohol consumption. Although this concept has been questioned for over 40 years, the review of methodologically reliable evidence data is scarce. This review summarizes the state of knowledge on the association between practicing sports and alcohol consumption among adolescents (10–19 years old) and its moderators. (2) Methods: The review covers only random-sample-based and population research. A systematic search was conducted on Scopus, PubMed, and WoS, for articles published between 2000 and 2021. From the 1944 identified records, 139 advanced to the full-text review, and 32 to the final data extraction and quality review. (3) Results: About two-thirds of the studies, including all the longitudinal ones, showed a positive association between sport participation and alcohol consumption. The most common mediators were gender (males were at higher risk), discipline (odds for team sports were higher, but professionalization could reduce it), and race, which intersected with gender, putting white males at the highest risk. (4) Conclusions: Further longitudinal research based on random samples using standardized indicators, including psychological and social variables, may provide more consistent outcomes and allow for the identification of mediating mechanisms.

## 1. Introduction

Alcohol consumption among adolescents is still common [1,2,3] and poses a serious threat to public health as harmful patterns of alcohol use cluster with other risk-taking behaviors and persist into adulthood [4]. Youths are particularly fragile to alcohol-related harm [5]. Exposure to alcohol at a young age leads to notable neural changes, resulting in limitations in cognitive functions, including verbal learning, attention, and visuospatial and memory tasks [6]. Alcohol consumption results in a decrement in educational achievements, increased absenteeism, and drop-out rates [7,8,9,10]. The deterrence hypothesis claims that alcohol consumption may be reduced by participation in sports, which provides youths with more constructive role models, supervision, increased bonds, and less need to express oneself in a destructive way [11]. Although this assumption started to be questioned in the 1980s and 1990s, the debate continues [12,13]. Research reviews show that sport participation is negatively associated with nicotine and illicit drug use, while for alcohol consumption, this association is positive [14,15]. This paper summarizes the current state of knowledge regarding the association between sport participation and alcohol consumption among adolescents (10–19 years old) and reveals the factors moderating the relationship between physical activity and alcohol consumption.

The triad of youth, sports, and alcohol has been the subject of many empirical studies, but the number of studies considering their synthesis is limited. All the available reviews on this topic involve research based on non-random samples [14,15,16,17,18]. However, the influence of the research design in a specific field is a subject of discussion [19]. By limiting the review to random-sample-based or population research and assessing the methodological quality of the papers, we hope to deliver evidence-based knowledge for substance overuse prevention and future research.

## 2. Materials and Methods

This scoping review followed the scoping review extension of Preferred Reporting Items for Systematic Review and Meta-Analysis (PRISMA) guidelines [20]. 

### 2.1. Search Strategy

We searched the Scopus, World of Science, and Medline (via PubMed) databases for the combinations and truncations of the following keywords: “youth*” OR “adolescent*” AND “sports” AND “alcohol”. The time frame was 1 January 2000 to 30 June 2021. The search returned 1944 records; after removing the 316 duplicates, 1628 were selected for the title and abstract screening. We used the Covidence software to facilitate this process.

#### Eligibility Criteria

- Examining the association between alcohol consumption (distinguished from other substances) and participation in sports (organized and/or not organized, competitive and/or recreational, and extracurricular school-based sports; papers covering school-based physical education were excluded);
- Original articles: reviews, letters, opinions, etc., were excluded;
- Quantitative research: qualitative projects were excluded;
- The researched population was aged 10–19. For longitudinal research, the first wave should fit the brackets. Grades were recoded based on age depending on country specifics;
- Random or population-based sample;
- Published in English.

After selection, 2 independent researchers performed the screening and a full review of 139 texts. Disagreements were solved through consensus. 

**Figure 1:**
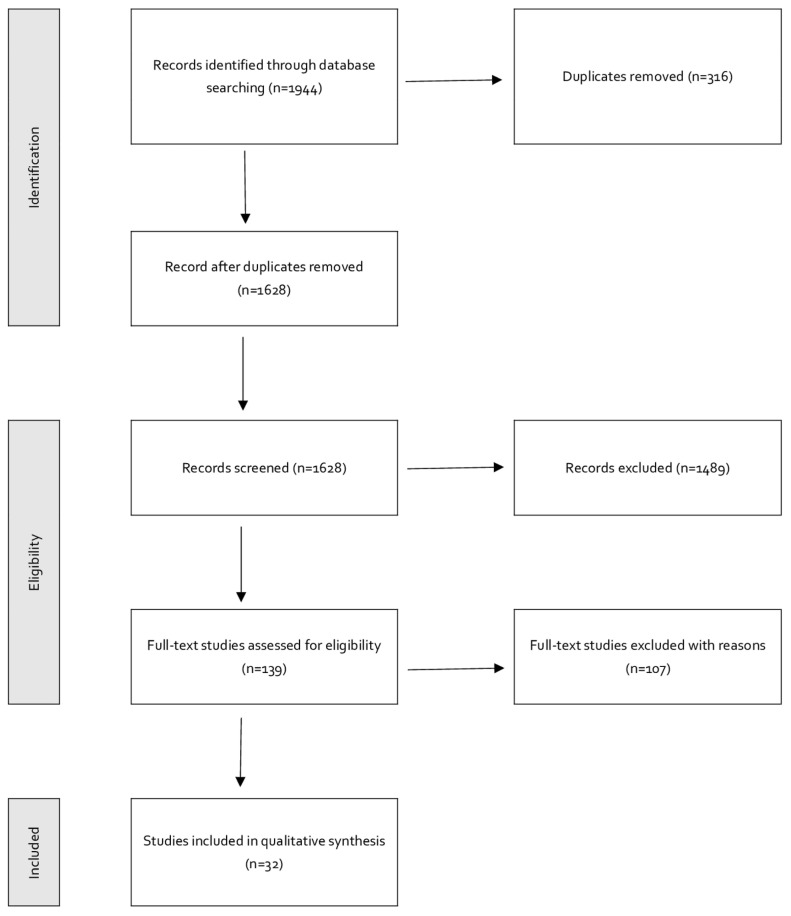
The PRISMA flowchart.

During the full-text review, we identified 49 papers that were based on non-random samples, 23 exceeded the assumed age brackets, and 8 were not in English. Seven papers were review articles, six were considered not adequate for the research question in this study, six did not distinguish sports from other extracurricular activities, and in five, alcohol was not differentiated from other substances. Three articles were not available. Finally, 32 papers were selected for quality control and data extraction (see Figure 1). 

### 2.2. Quality Control and Data Extraction

Quality control and data extraction were independently performed by two reviewers with predefined protocols, with disagreements solved through discussion. Four criteria were employed for quality control: (1) Does the study design allow for the evaluation of the association between sport participation and alcohol consumption? (2) Is the sample biased? (3) Are the alcohol consumption indicators reliable, precise, and adequate? (4) Are the indicators related to involvement in sports reliable, precise, and adequate? Each criterion was evaluated on a three-point scale: 1—poor, 2—moderate, and 3—strong. The general assessment involved the overall values computed as the share of the obtained points from the maximum sum of points (see Table 1).

All the correlation coefficients and odds ratios shown in the Results section were statistically significant (*p* < 0.05).

## 3. Results

### 3.1. Methodology 

Overall, 10 out of the 32 papers [26,27,28,31,32,33,36,45,49,51] solely focused on the relationships between involvement in sports and alcohol consumption. The others combined alcohol with other substances, but relevant data could be extracted. Over half (19 publications) of the articles originated from the USA, 3 from Brazil, and 2 from Spain. Other countries, namely Canada, Germany, France, Iceland, Japan, Kosovo, and Taiwan, were represented by one publication. One article [21] used the data obtained from thirty countries (see Table 2).

The most common research design, presented in 27 papers, was a cross-sectional study [21,22,23,24,26,27,29,31,32,33,35,36,37,38,39,40,41,42,43,44,45,46,47,48,49,50,51]. Five papers were based on longitudinal samples [11,25,28,30,34]. Two of the most common ways to measure sport activity were frequency (18 papers) [22,23,24,29,31,35,36,37,38,39,40,41,44,45,47,49,50,51] and the number of disciplines (14 publications) [11,21,26,28,31,33,35,38,41,43,44,45,48,50]. In five articles [25,27,32,34,42], a simple dichotomous variable (participation vs. non-participation) was used, three [23,30,51] used the intensity of involvement, and two [21,25] measured dichotomized school and out-of-school participation. Single papers included indicators of the duration of involvement in sport activity (in years), level of achievements, and being a club member [22,46]. Almost half of the studied publications (15 articles) distinguished different types of sports [11,22,26,28,29,31,32,33,35,39,40,41,43,44,45]. However, in four of them [11,28,41,45], this indicator was not used in the analysis (see Table 2).

Alcohol consumption was mostly measured by the frequency of consumption of at least one portion of alcohol within a defined period of time (24 papers) [23,24,25,26,27,28,29,30,32,33,35,36,38,39,40,41,42,43,44,45,48,49,50,51] and the frequency of heavy episode drinking, which included binge drinking (drinking 5 or more portions of alcohol in a row) or getting drunk (17 papers) [11,21,22,23,24,25,26,27,28,29,31,33,36,38,40,44,48]. In seven articles [24,27,34,37,38,40,50], the first exposure to alcohol was measured, while other indicators were only used in single projects: the quantity of alcohol [21,23]; standardized tools such as AUDIT [46] and KiGGS [40]; at least one exposition within a time period [47]; and drinking alcohol without parents’ knowledge [39] (see Table 2).

### 3.2. General Associations

More studies revealed a positive correlation between engagement in sports and alcohol use than those indicating a lack of significant association or negative relationship. Overall, 4 studies [31,34,43,44] reported a positive association between sport participation and alcohol consumption for the entire sample, while in other 15 studies [11,22,23,26,28,29,30,33,35,36,38,42,46,47,49], this positive association was observed for some fractions or under specific conditions. The longitudinal research by Mays and colleagues [28] revealed that participation in sports without other activities is associated with an increase in alcohol-oriented behaviors. Dever and co-authors [30] showed that when controlling for parental monitoring and school bonding, participation in sports was found to be a risk factor for alcohol use in males and low-risk-taking females. Other fractional and conditional cases are systematized and described in further sections.

In five papers [21,32,39,41,48], no significant associations were found, while two [25,27] reported ambiguous outcomes depending on gender: an increase in alcohol consumption among sportsmen and a decrease among sportswomen.

Sport participation resulted in a reduction in alcohol consumption in five studies [24,40,45,50,51]; however, the effect sizes were dispersed. To show two examples of the Spanish setting, Pastor and colleagues [24] reported a significant but weak negative correlation between participation in sports and alcohol consumption (R = −0.07). A study by Villalba and co-authors [45] showed a strong negative association between sport and physical activity and alcohol consumption (OR = 8.67 for boys and OR = 8.79 for girls). In comparison, Lee, Baek, and Nicholson [51] presented a more nuanced analysis. They indicated that even if participation in sports increases alcohol consumption, this effect is mediated through the development of self-esteem by practicing sports. Therefore, ultimately, sport participation reduces the risk. Chen and others [50] included community specifics, finding that participation in sports may reduce the risk of occasional drinking among youths living in areas with more options to buy alcohol. Diehl et al. [40] showed a reduction in alcohol use among elite athletes. In the research by Mays and Thompson [27], positive outcomes were found only for females.

### 3.3. Differences between Sports

Participation in ball games [52] is the most frequently associated with increased alcohol use among all sport groups. This correlation is, however, mediated by race and partially by gender. Veliz, Boyd, and McCabe [43] distinguished three groups of competitive sports: high-contact, semi-contact, and non-contact sports. Although adolescents who participated in competitive sports, in general, were more likely to get drunk within the last 30 days (OR = 1.477) and to experience early drunk episodes (OR = 1.237) than non-participants, the odds ratios for those practicing high-contact sports were higher (OR = 1.860 and OR = 1.741, respectively). Another sport with a positive association with alcohol consumption is weight training [44]. Peretti-Watel, Beck, and Legleye [22] found that gender differences moderated the results for different sports. Regular practice of athlete sports was negatively related to the repeated use of alcohol for girls and recent drunkenness for boys, while for girls, recent drunkenness correlated with strength and combat sports.

The negative influence of competitive team sport participation was reported by Lisha, Crano, and Delucchi [34]. By contrast, Adachi-Mejia and co-authors [39] found no significant correlations between participating in a team sport with a coach and drinking alcohol. The longitudinal research by Terry-McElrath, O’Malley, and Johnston [29] revealed that participating in athlete teams had no significant correlations with drinking and binge drinking in the last 30 days but was positively correlated with high school alcohol use. Exercise mitigates the negative influence of participation in team sports.

Additionally, differences were observed between team sports. Soccer players were more associated with heavy episodic drinking than their peers participating in other team sports, independently of their motivations to train [31]. An increase in alcohol consumption, including heavy episodic drinking among soccer players, was also reported in a paper by Bedendo and Noto [44]. On the other hand, Denham [35] found no significant impact of playing soccer on alcohol consumption. In his study, it was football that resulted in the increased frequency of alcohol consumption but only for white respondents.

### 3.4. Level of Involvement

Although high involvement increases the risk, it is mediated by the form of organization. Most of the studies found formally organized sport activity to be a protective factor. In their research, Halldorsson, Thorlindsson, and Sigfusdottir [36] found a negative correlation between the frequency of participation in formal sport and alcohol use (R = −0.147) and a positive, albeit extremely weak, association for informal sport participation (R = 0.036). The protective role of formal sports was confirmed in the study of Diehl et al. [40] on elite athletes, which revealed that elite athletes had a lower risk than non-elite athletes. Peretti-Watel, Beck, and Legleye [22] found that gender mediates between the intensity of sport activity and the repeated use of alcohol: the latter was reported less frequently among sportsmen undergoing moderate training (training up to 3 h a week, OR = 0.75) and more frequently among sportswomen undergoing intensive training (OR = 1.58). Interestingly, Stansfield showed a similar tendency but for male athletes performing high-intensity exercises [47]. On the other hand, two studies [38,49] found a higher level of consumption among frequently training athletes.

Another indicator of involvement was the number of disciplines practiced by the adolescents. Peretti-Watel, Beck, and Legleye [22] found that involvement in more than one sport increased the probability of the repeated use of alcohol for both sexes and the probability of recent drunkenness among boys. Vest and Simpkins [33] indicated that individuals involved in at least four disciplines reported a higher level of alcohol use, which, surprisingly, correlated with their athlete friends’ low consumption.

### 3.5. Club Participation

Most of the studies found no association between sports clubs participation and alcohol use, while one described clubs as channels of socialization that drive athletes toward alcohol consumption, with mediating role of professionalization. Studies by Peretti-Watel, Beck, and Legleye [22] and Fuijmoto and Valente [32] revealed that being registered as a sport club member does not correlate with alcohol consumption. In the research of Halldorsson, Thorlindsson, and Sigfusdottir [36], adolescents participating in formal sports were less likely to use alcohol than those participating in informal sports and non-participants, but the level of involvement mediated this association. Conversely, Fuijmoto and Valente [32] revealed that clubs may be channels for socialization: exposure to drinkers in a club results in a higher rate of alcohol consumption.

### 3.6. Gender

Gender visibly mediates the relationship between sport participation and alcohol use. Gender aspects were particularly considered in three studies [26,28,35], but no significant differences between males and females were observed. We will start by examining the studies showing significant results for women. Five papers [25,38,46,47,49] reported a positive association between sport participation and alcohol consumption exclusively among women. In the longitudinal research by Fredricks and Eccles [25], participation in high school sports was identified as a significant predictor of higher alcohol consumption one year after school for girls but not for boys. Dunn [38] revealed different patterns depending on the level of involvement. Women highly involved in recreational physical activity (five days a week or more) were more likely to have any past experiences with alcohol consumption, while those less frequently active had a higher odds ratio for drinking within the last 30 days. Both groups had higher indicators for binge drinking than non-participants. The probability of binge drinking increases with the number of sport disciplines. Other studies confirmed that high involvement among women goes along with a decrease in recent drinking. Tahiraj and colleagues [46] observed that the likelihood of risky alcohol consumption increased when the number of sport achievements increased among girls. Professionalization seems to play a role here: Stansfield [47] reported increasing alcohol consumption among women practicing less than four hours a day, but this tendency dissipated when daily training reached four hours. Similarly, while King et al. [49] reported increased odds for recent alcohol consumption and past alcohol consumption among all females, they revealed decreased ratio among frequently practicing athletes.

Two studies reported lower exposure to alcohol among sportswomen. Female athletes, contrary to males, were less likely to have first drunk at age 12 or earlier (OR = 0.824), to have drunk recently (OR = 0.843), and to have ever drunk (OR = 0.807) [27]. Similar conclusions were drawn by Guèvremont and co-authors [37]. Finally, only one study [30] did not reveal any influence (contrary to boys), with the exception of lower alcohol consumption in the fraction of low-risk-taking female students in the 8th grade.

The association between alcohol consumption and sport participation among male adolescents was reported in nine studies [11,26,27,30,35,38,42,47,49], although in some of them, only for specific fractions. Participation in sports was a significant predictor of alcohol use in the research conducted by Dever and colleagues [30]. The association was stronger for older (10th graders), high-risk-taking males [30]. Males involved in sports but without other activities had a higher rate of alcohol use than their non-athlete peers [23,47]. Takakura [42] reported a positive association between participation in a community sport club and drinking alcohol among boys. Mays and Thompson [27] found that the risk of the initiation of alcohol use at a young age is generally lower among athletes (OR = 0.704), but male athletes are more likely to report heavy drinking than non-athlete peers (OR = 1.204). By contrast, two studies [38,49] reported that males involved in recreational sports were more likely to ever try alcohol and report recent drinking. In their longitudinal study, Fredricks and Eccles [25] reported that sport participation may be related to lower alcohol use for boys (the exact difference was not reported). This association becomes insignificant one year after high school.

### 3.7. Race

Evidence on race as a mediator is limited and has emerged only from the USA. They show, however, a significant role of race mediated by discipline. Eitle and colleagues [11] found that participation in sports correlates with alcohol use among white males, especially playing football in the 12th grade. A similar influence was also found on white males playing basketball and football as well as white female basketball players in Denham’s study [35]. Fredricks and Eccles [25] found that African American youths reported drinking less than their European American peers.

## 4. Discussion

Most of the examined studies, that is 21 out of 32, including all longitudinal studies, revealed a positive association between sport involvement and alcohol consumption. Considering the fact that five other studies found no correlation, this review sheds light on another argument against the deterrence hypothesis. There are several limitations, however. Transnational comparisons are prone to cultural differences, which may play a role in consumption patterns [53]. If we limit to the US-based studies, proportions will slightly alter: Notably, 12 papers revealed a negative influence, 4 revealed no association, 2 studies revealed an ambiguous correlation, and in one study, a decrease in alcohol use was observed. Europe-based studies are scarce, and the conclusions are ambiguous. Three showed a negative association, while another three studies revealed a positive association; however, one of the latter was focused on elite athletes only. Considering the small number of studies from culturally homogenous areas, any conclusions are weak, but changes in proportions clearly highlight the role of cultural patterns.

Moreover, while the indicators used by the majority of authors to measure the level of consumption are coherent, there are significant differences in the measurement of sport involvement. A surprisingly low number of studies used standardized tools such as PAQ-A [54]. Survey-based studies are also vulnerable to uncontrolled respondent bias. However, probably the most significant methodological limitation is to derive conclusions from correlational analyses. We were able to identify only five longitudinal studies that overcome this limitation.

Two studies showed no differences between athletes in team sports and those in non-team sports [39,44]. The negative influence of participation in team sports [29] is mediated by the level of involvement and form of organization. It seems that professionalization, to some extent, reduces the potentially negative socialization patterns obtained in peer groups and limited parental control. Our review does not reveal evidence to support or neglect the U hypothesis [55], as the results are ambiguous. This can also be attributed to specific cultural factors.

Evidence on differences between particular disciplines is limited, as few studies only identified specific sports. Most of the proofs of association between sport involvement and alcohol use involve ball games [22,29,31,43], including football, soccer, ice hockey, and lacrosse [52]. One study found an increase in alcohol consumption among power and weight-dependent game athletes [22], while no association was found for aesthetic sports (diving) [44], and a decrease was observed for technical and endurance sports [22]. It seems that differences between sports may come not from the specifics of the discipline, but the way the training is managed and how the coaches mediate the possible influence of peers. The second possible explanation is unequal gender [22] and race [31] distribution among athletes. Thirdly, cultural differences may play a significant role. For example, two studies showing a higher consumption ratio among soccer players [31,44] come from Brazil, while the one that found no association is US-based [35]. The cultural patterns connected with soccer and the social selection of players are probably different in these two countries.

The majority of studies reported gender effects. The negative influence of sport participation was reported more frequently for males (nine studies revealed a positive association, while in one study, a negative association was found) than for females (five to one, respectively). This difference may result from the specific attributes of male/female peer group cultures [25]. However, four studies, comprising three from the USA and one multinational, found no differences between genders. The evidence on the role of race, however, is limited to only US-based studies, which highlight the intersection of gender and race. The group of athletes at risk are white males, in particular those playing team sports. This can be attributed to the different patterns of alcohol consumption in social groups [25] and the fact that sports may contribute to social advancement for underprivileged groups.

### 4.1. Practical Implications

This review strengthens the conclusion that sports alone are not preventive mechanisms against alcohol use by youths and allows the identification of the most vulnerable groups. The results highlight the challenges faced by physical education teachers, coaches of school sport teams, and coaches in sport clubs if the sport is to have a preventive function. Preventive interventions can bring positive effects, as shown by the results of an experiment using individual health consultations for students practicing sports [56]. However, as indicated by Ng and co-authors [57], sport coaches discuss psychoactive substances as a reactive strategy to the consumption of alcohol by young athletes. The urgent need to promote health among those who practice sports appears to be related to an improvement in management strategies in that teachers and sport coaches need to implement preventive measures against alcohol consumption among young athletes.

### 4.2. Limitations

A number of limitations can be identified in this review. No studies published outside peer-reviewed journals or the grey literature were included, and studies other than English-speaking publications were not considered. Qualitative studies were also excluded concerning the aim of this review. The selected studies are not equally dispersed geographically, with a significant over-representation of US-based studies. The few studies from other areas (Europe and Asia) make any comparisons between the regions suspect. The fact that all the studies enclosing race as a mediator are only from the USA makes any generalizations about the role of race questionable.

The rapid research in other databases (Biomed, Central BioOne, BIOSIS, EBSCO, JSTOR, ProQuest, SAGE, Science Direct, SpringerLink, Tylor, and Francis and Wiley) brought no additional studies meeting the eligibility criteria, but the results of this review may be affected by the selection of databases.

## 5. Conclusions

Although the majority of the examined studies concluded that alcohol consumption and engagement in sports are positively correlated, more research is required. From the methodological point of view, longitudinal random-sample-based studies using standardized indicators provide more consistent conclusions. Areas other than the USA seem to be under-researched, and more outcomes from different countries would allow intercultural comparisons and estimation of the influence of different socialization patterns. It would also be useful to enclose race as a mediator in non-US-based studies. As for the future direction of this research, more psychological and social variables should be included to identify the mechanism mediating alcohol consumption in sport settings.

## Figures and Tables

**Table 1:** Quality assessment details.

Article	Study Design	Sample External Validity	Alcohol Use Indicators	Sport Involvement Indicators	Overall Assessment (100% = 12 Points)
Pate et al., 2000 [21]	2	3	2	1	67%
Peretti-Watel et al., 2002 [22]	2	3	1	3	75%
Eilte et al., 2003 [11]	3	2	1	2	67%
Harrison, Narayan, 2003 [23]	2	3	3	3	92%
Pastor et al., 2003 [24]	2	3	3	2	83%
Fredricks and Eccles, 2006 [25]	3	3	3	1	83%
Hoffmann, 2006 [26]	2	3	3	2	83%
Mays and Thompson, 2009 [27]	2	3	3	1	75%
Mays et al., 2010 [28]	3	3	3	2	92%
Terry-McElrath et al., 2011 [29]	2	3	3	3	92%
Dever et al., 2012 [30]	3	3	2	2	83%
Bedendo et al., 2013 [31]	3	2	2	3	83%
Fuijmoto and Valente, 2013 [32]	2	3	2	2	75%
Vest and Simpkins, 2013 [33]	2	3	3	2	83%
Lisha et al., 2014 [34]	2	3	1	1	58%
Denham, 2014 [35]	2	3	2	3	83%
Halldorsson et al., 2014 [36]	2	3	3	2	83%
Guèvremont et al., 2014 [37]	2	3	1	2	67%
Dunn, 2014 [38]	2	3	3	3	92%
Adachi-Mejia et al., 2014 [39]	2	3	2	3	83%
Diehl et al., 2014 [40]	2	3	3	3	83%
Silva et al., 2014 [41]	2	3	2	3	83%
Takakura, 2015 [42]	2	3	2	1	67%
Veliz et al., 2015 [43]	2	3	2	2	75%
Bedendo and Noto 2015 [44]	2	2	3	3	83%
Villalba et al., 2016 [45]	2	3	2	3	83%
Tahiraj et al., 2016 [46]	2	3	3	2	83%
Stansfield, 2017 [47]	2	3	1	2	67%
Lane and Decamp, 2017 [48]	2	3	2	1	67%
King et al., 2017 [49]	2	2	2	2	67%
Chen et al., 2019 [50]	2	2	1	3	67%
Lee et al., 2020 [51]	2	3	3	2	83%

Scale: 1—poor, 2—moderate, and 3—strong.

**Table 2:** The location, methodology, and main outcomes from reviewed studies.

Study	Country	Study Design	Indicators of Alcohol Consumption					Indicators of Sport Participation				Main Conclusions
			Quantity	Frequency	HED ^A^	Initiation	Other	Frequency	No of Disciplines	Disciplines Identification	Other	
Pate et al., 2000 [21]	USA	cross-sectional	X		X				X		X ^F^	No association
Peretti-Watel et al., 2002 [22]	France	cross-sectional			X			X		X	X ^J^	A weak association for sportsmen performing moderate training and a stronger association for sportswomen performing intensive training. Regular practice of athlete sports correlates with a decrement in AC for females and an increment for males. Females practicing strength and combat sports have a higher AC level. Involvement in more than one sport increases AC. No influence of club membership.
Eilte et al., 2003 [11]	USA	longitudinal			X				X	X ^D^		No general association, but higher AC was observed among white males.
Harrison, Narayan, 2003 [23]	USA	cross-sectional	X	X	X			X			X ^G^	Positive association for males
Pastor et al., 2003 [24]	Spain	cross-sectional		X	X	X		X				Small negative correlation
Fredricks and Eccles, 2006 [25]	USA	longitudinal		X	X						X ^FH^	Lower AC and later alcohol initiation among male SP Reverse outcomes for girls
Hoffmann, 2006 [26]	USA	cross-sectional		X	X				X	X		Positive correlation. A stronger association between females in lower SES schools and males in higher SES schools
Mays and Thompson, 2009 [27]	USA	cross-sectional		X	X	X					X ^H^	Positive correlation for males; female SP have lower AC.
Mays et al., 2010 [28]	USA	longitudinal		X	X				X	X ^D^		Positive correlation for both genders
Terry-McElrath et al., 2011 [29]	USA	cross-sectional study		X	X			X		X		No correlation of SP with recent AC, but SP increases the probability of ever AC.
Dever et al., 2012 [30]	USA	longitudinal study		X							X ^G^	SP increases AC among males, especially for high-risk older students (10th graders). No associations for females, with the exception of low risk-taking younger students (8th grade) SP who have lower AC levels.
Bedendo et al., 2013 [31]	Brazil	cross-sectional study			X			X	X	X		Higher AC among soccer players than other team sports
Fuijmoto and Valente, 2013 [32]	USA	cross-sectional		X						X	X ^H^	No influence of club membership
Vest and Simpkins, 2013 [33]	USA	cross-sectional		X	X				X	X		Positive correlation; no influence of participation in multiple sports. Peers’ patterns of AC have a strong influence on individual AC.
Lisha et al., 2014 [34]	USA	longitudinal				X					X ^H^	Negative correlation for participation in team sports
Denham, 2014 [35]	USA	cross-sectional		X				X	X	X		No correlation for soccer players; a positive correlation for white football and basketball male players and white basketball female players
Halldorsson et al., 2014 [36]	Iceland	cross-sectional		X	X			X				Higher SP results in higher AC among athletes not registered in sport clubs; however, the latter have a higher level of AC than non-participants.
Guèvremont et al., 2014 [37]	Canada	cross-sectional				X		X				Positive association for males and negative for females.
Dunn, 2014 [38]	USA	cross-sectional		X	X	X		X	X			Positive association. HED ^A^ increases with the number of disciplines. High SP reduces alcohol initiation among females.
Adachi-Mejia et al., 2014 [39]	USA	cross-sectional		X				X		X		No correlation between participating in team sports with a coach and AC
Diehl et al., 2014 [40]	Germany	cross-sectional		X	X	X	KIGGS	X		X		Lower AC among elite athletes than among non-elite
Silva et al., 2014 [41]	Brazil	cross-sectional		X				X	X	X ^E^	PAQ-A	No influence
Takakura, 2015 [42]	Japan	cross-sectional		X							X ^H^	Positive association for males
Veliz et al., 2015 [43]	USA	cross-sectional		X					X	X		Positive correlation
Bedendo and Noto, 2015 [44]	Brazil	cross-sectional		X	X			X	X	X		Higher AC among soccer players and weightlifters
Villalba et al., 2016 [45]	Spain	cross-sectional		X				X	X	X ^E^	PAQ-A	Strong negative association for males and females
Tahiraj et al., 2016 [46]	Kosovo	cross-sectional					AUDIT				X ^I^	Positive correlation between AC and sport achievements
Stansfield, 2017 [47]	^B^	cross-sectional					X ^C^	X				Intense SP results in lower AC than moderate or low sport activity Low and moderate involvement increases AC
Lane and Decamp, 2017 [48]	USA	cross-sectional		X	X				X			Positive association
King et al., 2017 [49]	USA	cross-sectional		X				X				Positive correlation for male athletes. Access to alcohol is positively correlated with the intensity of SP. Non-frequently practicing women have higher AC, while frequently practicing sportswomen have lower AC.
Chen et al., 2019 [50]	Taiwan	cross-sectional	X ^C^			X		X	X			SP reduces AC but only in specific communities. Access to alcohol is positively correlated with the intensity of SP.
Lee et al., 2020 [51]	USA	cross-sectional		X				X			X ^G^	SP increases AC, but increasing self-esteem mediates the effect. Finally, SP reduces the risk.

Notes: RS—random sample, PS—population study, AC—alcohol consumption, SP—sport participation. ^A^ Heavy episodes of drinking, binge drinking, drinking five or more portions of alcohol in a row, getting drunk. ^B^ Cyprus, United States, Aruba, Portugal, Iceland, Russia, Armenia, Netherlands, Sweden, Austria, Bosnia-Hercegovina, Ireland, Poland, Slovenia, France, Lithuania, Hungary, Switzerland, Spain, Canada, Venezuela, Czech Republic, Belgium, Norway, Finland, Denmark, Italy, Netherlands Suriname, Germany, and Estonia. ^C^ At least a single occurrence within a time period. ^D^ Identified but not used in the analysis. ^E^ PAQ-A differentiates disciplines, but it was not used in the analysis. ^F^ In-school vs. out-of-school activities. ^G^ Advance level. ^H^ Dichotomous indicator. ^I^ Length of athlete’s career and sport achievements. ^J^ Club registration.

## Data Availability

Not applicable.
